# Ultraviolet-ozone-treated PEDOT:PSS as anode buffer layer for organic solar cells

**DOI:** 10.1186/1556-276X-7-465

**Published:** 2012-08-17

**Authors:** Zisheng Su, Lidan Wang, Yantao Li, Haifeng Zhao, Bei Chu, Wenlian Li

**Affiliations:** 1 State Key Laboratory of Luminescence and Applications, Changchun Institute of Optics, Fine Mechanics and Physics, Chinese Academy of Sciences, Changchun, 130033, People’s Republic of China

**Keywords:** Organic solar cell, PEDOT:PSS, UV-ozone

## Abstract

Ultraviolet-ozone-treated poly(3,4-ethylenedioxythiophene):poly(styrene sulfonate) (PEDOT:PSS)was used as the anode buffer layer in copper phthalocyanine (CuPc)/fullerene-based solar cells. The power conversion efficiency of the cells with appropriated UV-ozone treatment was found to increase about 20% compared to the reference cell. The improved performance is attributed to the increased work function of the PEDOT:PSS layer, which improves the contact condition between PEDOT:PSS and CuPc, hence increasing the extraction efficiency of the photogenerated holes and decreasing the recombination probability of holes and electrons in the active organic layers.

## Background

Organic solar cells (OSCs) have attracted significant interests because of their potential for renewable energy source, low-cost and large-scale fabrication, and compatibility with large-area and flexible substrates [1]. In the past two decades, the power conversion efficiency (PCE) of OSCs has been steadily improved, and a PCE exceeding 8% has been demonstrated by using the materials that exhibit a broad absorption with high coefficient in the solar spectrum and by developing new device configurations that provide high exciton dissociation efficiency and charge carrier collection efficiency [2,3]. The mechanism of OSCs involves the formation of excitons under illumination, the diffusion of excitons to the donor-acceptor interface, the dissociation of excitons into electrons and holes, and the collection of electrons and holes at opposite electrodes. One of the most important factors in determining the charge carrier collection efficiency is the interface property of electrode/organic layer. The buffer layer is often adopted in OSCs to improve the device performance. A lot of anode buffer layers have been demonstrated, such as MoO_3_[4], V_2_O_5_[4], NiO [5], WO_3_[6], and graphene oxide [7]. Conductive poly(3,4-ethylenedioxythiophene):poly(styrene sulfonate) (PEDOT:PSS) film presents many advantages, such as high transparency in the visible range, high mechanical flexibility, and excellent thermal stability. These properties make it beneficial to be used as the anode buffer layer in OSCs [8]. However, phase separation of PEDOT and PSS is generally found with the insulating PSS grains atop the as-prepared PEDOT: PSS film cast from aqueous PEDOT:PSS solution, which leads to a low conductivity below 1 S/cm and an improper contact condition between PEDOT:PSS and the following organic layer [9]. Many strategies have been proposed to address such an issue, such as adding sorbitol [10-12], glycerol [11], N-methylpyrrolidone [12], isopropanol [12], dimethyl sulfoxide [13,14], N,N-dimethyl formamide [13], tetrahydrofuran [13], ethylene glycol [14], 2-nitroethanol [14], 1-methyl-2-pyrrolidinone [14], mannitol [15], sodium p-toluenesulfonate [16], carbon nanotube [17], and pentacene [18] into PEDOT:PSS aqueous solution and treating the as-prepared PEDOT:PSS film with solvents [14,19], thermal annealing [20], oxygen plasma [21], Ar ion sputtering [22], zwitterions [23], salt solution [24], and H_2_SO_4_[25]. However, these methods make the device construction process more complex and require careful control of the technologies to avoid the deterioration of the PEDOT:PSS film properties.

Increased work function and conductivity of PEDOT:PSS film have been demonstrated by ultraviolet light irradiation [26,27], and the treated PEDOT:PSS has been adopted as the anode buffer layer in OSCs [28,29]. Tengstedt et al. [30] have proposed that the work function of PEDOT:PSS film can be increased while maintaining reasonable conductivity by UV-ozone treatment, which is further confirmed by Helander et al. [31]. Nagata et al. [32] have clarified the respective roles of UV light irradiation and exposure to ozone gas on the PEDOT:PSS film, and they have found that the main role of UV light is to decompose the chemical bonds in the PEDOT:PSS film, resulting in a decrease of the conductivity, while the ozone and atomic oxygen are absorbed and oxidize the surface, leading to an increase of the work function. Thus, the UV-ozone treatment is capable of controlling the work function and conductivity of PEDOT:PSS film, hence allowing them to be adjusted to the device application. Such UV-ozone-treated PEDOT:PSS film has been adopted as the anode buffer layer in organic light-emitting diodes, and dramatic improvement of efficiency was observed [33,34]. However, the application of UV-ozone-treated PEDOT:PSS in OSCs has not been exploited. In this paper, UV-ozone-treated PEDOT:PSS film is adopted as the anode buffer layer in copper phthalocyanine (CuPc)/fullerene (C_60_)-based small molecular OSCs. The power conversion efficiency of the cell was increased by more than 20%, compared with the reference cell without UV-ozone treatment. The improvement is primarily attributed to the increased work function of the PEDOT:PSS film, which improves the contact condition between PEDOT:PSS and CuPc, hence increasing the charge carrier collection efficiency and decreasing the charge carrier recombination probability in the bulk of organic layers.

## Methods

Devices were fabricated on pattered indium tin oxide (ITO)-coated glass substrates with a sheet resistance of 15 Ω/sq. The substrates were routinely cleaned, followed by UV-ozone treatment for 10 min. The structure of the OSCs used here was ITO/PEDOT:PSS/CuPc (30 nm)/C_60_(40 nm)/4,7-diphenyl-1,10-phenanthroline (8 nm)/Al (100 nm). Two types of PEDOT:PSS (Clevios P VP Al 4083 (H. C. Starck, Clevios GmbH, Leverkusen, Germany) and 483095 (Aldrich, St. Louis, MO, USA) with PEDOT/PSS mass ratio of 1:6 and 1:1.6, respectively) were used here, and they were spin-coated onto the ITO anode with a speed of 4,000 rad/min, followed by baking in vacuum at 120 °C for 1 h, which forms a PEDOT:PSS layer of about 30 nm. The PEDOT:PSS films were then treated in a UV-ozone environment for different times(0, 2, 4, 6, and 10 min) before loading into a high-vacuum chamber. The other organic layers and the cathode were deposited onto the substrates via thermal evaporation in the vacuum chamber at a pressure of approximately 10^−7^ Torr. Deposition rates and thickness of the layers were monitored *in situ* using oscillating quartz monitors. The evaporation rates were kept at approximately 1 Å/s for organic layers and Al cathode. Current–voltage (*I*-*V*) characteristics of the devices were measured with a programmable Keithley 2400 power source (Keithley Instruments, Inc., Cleveland, OH, USA) both in dark and under illumination of a Xe lamp light source with an intensity of 100 mW/cm^2^. The surface characterization of PEDOT:PSS films was performed with a Bruker MultiMode 8 atomic force microscope (AFM; BRUKER, Ettlingen, Germany) in tapping mode. All the measurements were carried out at room temperature under ambient conditions.

## Results and discussion

**Figure 1:**
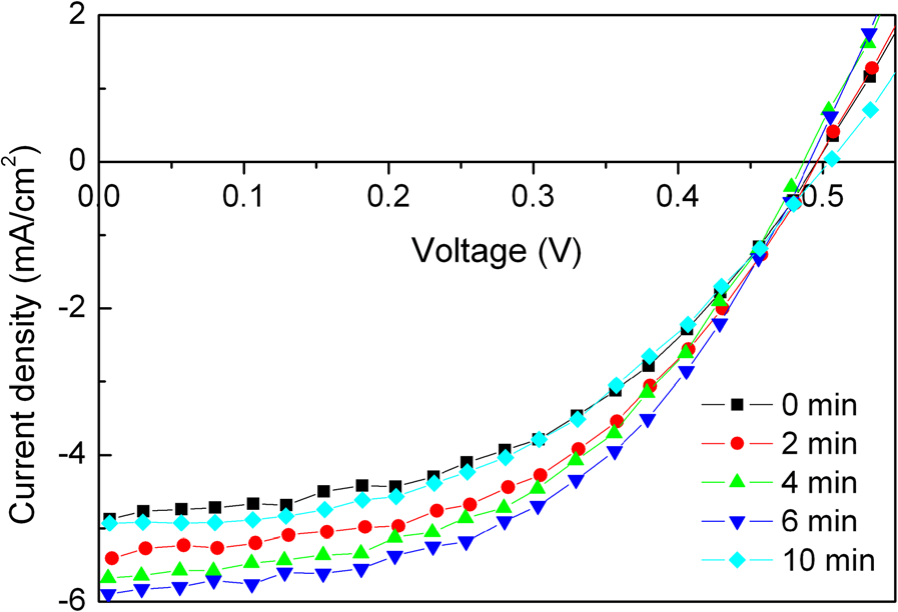
*** I*-*V *characteristics of the cells with PEDOT:PSS (Clevios P VP Al 4083) anode buffer layer.** Treated with UV-ozone for various times under illumination.

Figure 1 shows the *IV* characteristics of the cells under illumination with a PEDOT:PSS (Clevios P VP Al 4083) anode buffer layer treated with UV-ozone for various times. The parameters extracted from the *IV* curves are summarized in Table 1. The reference cell with untreated PEDOT:PSS film shows an open circuit voltage (*V*_OC_), short-circuit current density (*J*_SC_), fill factor (FF), and PCE of 0.496 V, 4.872 mA/cm^2^, 0.477, and 1.149%, respectively. It can be found in Figure 1 and Table 1 that the *V*_OC_ of the cells with PEDOT:PSS anode buffer layer is almost constant regardless of the UV-ozone treatment time. The *V*_OC_ of the CuPc/C_60_-based OSCs is reported to be determined by the energy offset of the highest occupied CuPc molecular orbital and the lowest unoccupied C_60_ molecular orbital [35]. The same *V*_OC_ of the cells suggests that the UV-ozone treatment on PEDOT:PSS does not alter this energy offset. In contrast, the *J*_SC_ and FF of the cells first increase and then decrease with further increase of the UV-ozone treatment time. The cell with 6-min UV-ozone-treated PEDOT:PSS shows the maximum *J*_SC_ and FF of 5.897 mA/cm^2^ and 0.495, respectively. Consequently, a PCE of 1.429% was obtained, which was increased by 24% compared to the reference cell. 

**Table 1:** **Performance of cells with** Clevios **P VP Al 4083 PEDOT:PSS anode buffer layer**

**UV treatment time (min)**	***V***_**OC**_	***J***_**SC**_	**FF**	**PCE**
	**(V)**	**(mA/cm**^**2**^**)**		**(%)**
0	0.496	4.872	0.477	1.149
2	0.496	5.407	0.486	1.303
4	0.487	5.675	0.489	1.351
6	0.491	5.897	0.495	1.429
10	0.504	4.931	0.466	1.160

Performance of the cells under illumination with a PEDOT:PSS (Clevios P VP Al 4083) anode buffer layer treated with UV-ozone for various times. FF, fill factor; PCE, power conversion efficiency.

**Figure 2:**
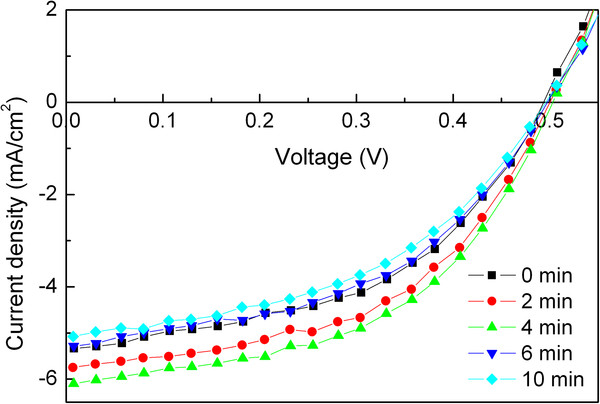
*** I*-*V *characteristics of the cells with PEDOT:PSS (Aldrich 483095) anode buffer layer.** Treated with UV-ozone for various times under illumination.

Figure 2 describes the *I*-*V* characteristics of the cells under illumination with a PEDOT:PSS (Aldrich 483095) anode buffer layer treated with UV-ozone for various times. The parameters extracted from the *I*-*V* curves are listed in Table 2. The *V*_OC_, *J*_SC_, FF, and PCE of the reference cell are 0.493 V, 5.334 mA/cm^2^, 0.483, and 1.271%, respectively. The superior performance of the cell with Aldrich 483095 PEDOT:PSS compared with Baytron P VP Al 4083 PEDOT:PSS is attributed to the higher conductivity of the former, which may increase the extraction efficiency of the photogenerated charge carriers. Similarly, the *V*_OC_ of the cells are unaffected by the UV-ozone treatment on the PEDOT:PSS layer. In contrast, the *J*_SC_ and FF are increased with UV-ozone treatment time. The maximum *J*_SC_ and FF are found with 4-min UV-ozone treatment time, which are 6.099 mA/cm^2^ and 0.499, respectively. Correspondingly, the PCE reaches up to 1.529%, which is increased by 20% compared to the reference cell. These findings suggest that the improved performance of the CuPc/C_60_-based OSCs can be observed with UV-ozone-treated PEDOT:PSS as the anode buffer layer, and this effect is valid for the PEDOT:PSS film with different conductivities. 

**Table 2:** Performance of the cells with Aldrich 483095PEDOT:PSS anode buffer layer It is appropriate

**UV treatment time (min)**	***V***_**OC**_	***J***_**SC**_	**FF**	**PCE**
	**(V)**	**(mA/cm**^**2**^**)**		**(%)**
0	0.493	5.334	0.483	1.271
2	0.499	5.745	0.504	1.446
4	0.502	6.099	0.499	1.529
6	0.497	5.282	0.472	1.240
10	0.495	5.074	0.459	1.059

Performance of the cells under illumination with a PEDOT:PSS (Aldrich 483095) anode buffer layer treated with UV-ozone for various times. FF, fill factor; PCE, power conversion efficiency.

**Figure 3:**
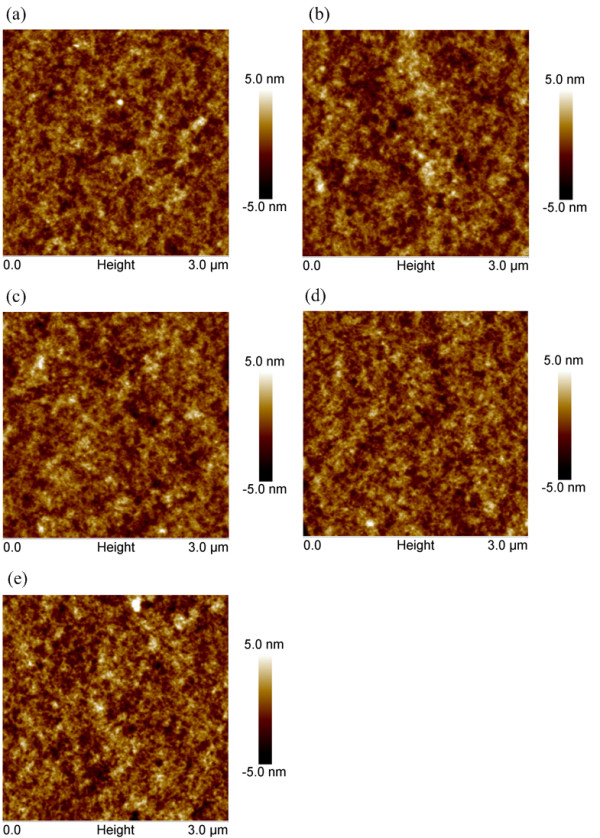
**AFM images of the ITO/PEDOT:PSS (CleviosP VP Al 4083) films.** Treated with various times: (**a**) 0, (**b**) 2, (**c**) 4, (**d**) 6, and (**e**) 10 min.

The improvement of the device performance by UV-ozone treatment may come from two factors: improved interface contact condition in PEDOT:PSS/CuPc and increased conductivity of the PEDOT:PSS film. Figure 3 displays the AFM image of the PEDOT:PSS (Clevios P VP Al 4083) films on ITO anode treated with UV-ozone for various times. The morphology of the PEDOT:PSS film without UV-ozone treatment is quite smooth with a root mean square (RMS) roughness of 1.06 nm, while the RMS roughnesses of the 2-, 4-, 6-, and 10-min UV-ozone-treated PEDOT:PSS films are 1.15, 1.10, 1.08, and 1.23 nm, respectively. This finding indicates that the UV-ozone treatment has little effect on the morphology of the PEDOT:PSS films. Thus, the PEDOT:PSS morphology change-induced alterations of the optical field distribution in the active organic layers, the CuPc molecule stacking mode, and the crystallinity of CuPc layer could be ruled out for the improved performance of the OSCs. Tengstedt et al. [30] have found that both the PEDOT and PSS moistures of the PEDOT:PSS film could be oxidized under UV-ozone treatment, which results in an increase of the work function of the PEDOT:PSS film. The effect was further confirmed by Helander et al. [31] and Nagata et al. [32]. Thus, increased work function of PEDOT:PSS film with UV-ozone treatment can be expected in this study. Such an effect improves the contact condition between PEDOT:PSS and CuPc, which increases the extraction efficiency of the photogenerated holes and decreases the recombination probability of holes and electrons in the active organic layers. As a result, both the *J*_SC_ and FF and, hence, the PCE are enhanced in the optimized cells. 

**Figure 4:**
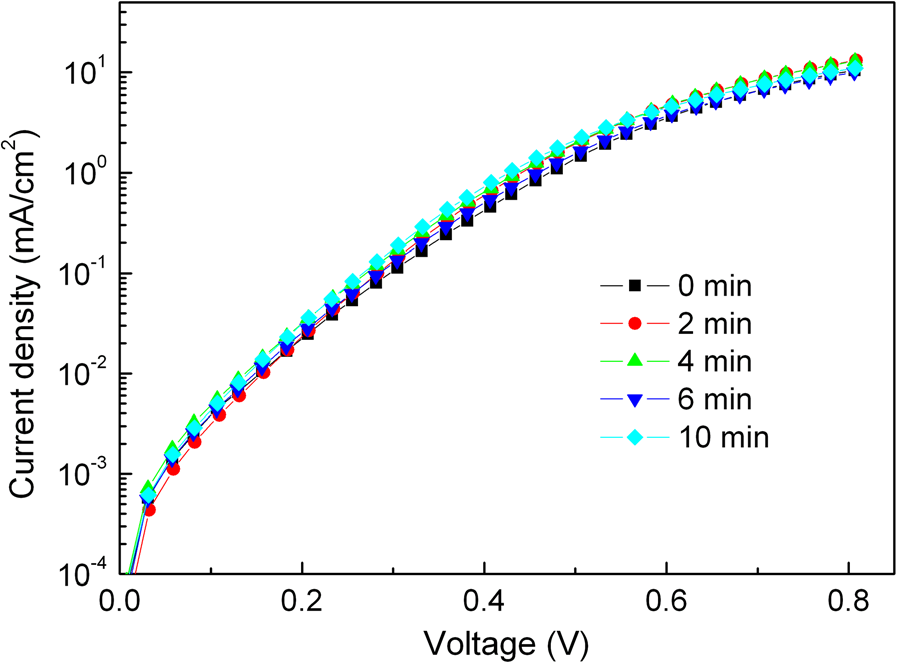
**Dark current density of cells with PEDOT:PSS (CleviosP VP Al 4083) anode buffer layer.** Treated with UV-ozone for various times.

To exploit the UV-ozone treatment on the conductivity of the PEDOT:PSS film, the dark current of the cells with a PEDOT:PSS (Clevios P VP Al 4083) anode buffer layer treated with UV-ozone for various times was investigated, as shown in Figure 4. All five cells present almost the same the *IV* curves. A similar phenomenon was found in the cells with Aldrich 483095 PEDOT:PSS as the anode buffer layer (not shown here). Such a fact indicates that the UV-ozone treatment has little affect on the conductivity of the PEDOT:PSS film during the time scale investigated. Furthermore, this finding rules out the contribution of the increased conductivity of the PEDOT:PSS layer to the improved device performance. Thus, the improved device performance is attributed to the increased work function of the PEDOT:PSS layer under UV-ozone treatment, which increases the extraction efficiency of the photogenerated holes and decreases the recombination probability of holes and electrons in the active organic layers. The decreased performance of the cells with prolonged UV-ozone treatment time may result from the saturation in the change of the work function, decomposition of the chemical bonds, and/or formation of ping hole defects in the PEDOT:PSS layer [32,33], which would decrease the extraction efficiency of the photogenerated charge carriers. 

## Conclusions

In summary, UV-ozone-treated PEDOT:PSS film was used as the anode buffer layer in CuPc/C_60_-based OSCs. The morphology of the PEDOT:PSS film is unaffected by the UV-ozone treatment. However, the PCE is found to increase about 20% compared to the reference cell without UV-ozone treatment. The improved performance is attributed to the increased work function of the PEDOT:PSS layer, which increases the extraction efficiency of the photogenerated holes and decreases the recombination probability of holes and electrons in the active organic layers. This work provides a facile and cost-effective method to improve the performance of OSCs. Besides, such a strategy may have potential applications to improve the contact condition between PEDOT:PSS and metal anode in inverted OSCs where a PEDOT:PSS/metal bilayer anode is adopted.

## Competing interests

The authors declare that they have no competing interests.

## Authors’ contributions

ZS participated in the design of the study, carried out the experiments, collected data, performed data analysis, and drafted the manuscript. LW and YL participated in collection of the data, performed data analysis, and helped draft the manuscript. HZ participated in the AFM image measurement and analysis. BC and WL participated in the design of the study and helped draft the manuscript. All authors read and approved the final manuscript.
